# Association of child neurodevelopmental or behavioural problems with maternal unemployment in a population-based birth cohort

**DOI:** 10.1007/s00127-023-02464-6

**Published:** 2023-03-26

**Authors:** Joana Amaro, Raquel Costa, Maja Popovic, Milena Maria Maule, Ingrid Sivesind Mehlum, Raquel Lucas

**Affiliations:** 1 EPIUnit - Instituto de Saúde Pública, Universidade do Porto, Rua das Taipas, nº 135, 4050-600 Porto, Portugal; 2 Laboratório para a Investigação Integrativa e Translacional em Saúde Populacional (ITR), Rua das Taipas, nº 135, 4050-600 Porto, Portugal; 3 Department of Public Health and Forensic Sciences, and Medical Education, University of Porto Medical School, Porto, Portugal; 4 Digital Human-Environment Interaction Lab (HEI-Lab), Universidade Lusófona, Lisbon, Portugal; 5 Unit of Cancer Epidemiology, Department of Medical Sciences, University of Turin and CPO Piemonte, Turin, Italy; 6 Department of Occupational Medicine and Epidemiology, National Institute for Occupational Health, Oslo, Norway

**Keywords:** Child health, Cohort study, Employment, Neurodevelopmental disorders

## Abstract

**Purpose:**

To estimate associations between suspected or diagnosed neurodevelopmental or behavioural problems in 7-year-old children and maternal unemployment at child age 7 and 10, in a Portuguese birth cohort.

**Methods:**

We evaluated 5754 mothers and their children of the population-based birth cohort Generation XXI in Porto, Portugal. Data on suspected and diagnosed child neurodevelopmental and behavioural problems (exposures)—learning, attention and language problems, externalising behaviours, developmental delay, autism spectrum disorders, and other neurodevelopmental problems—were retrieved at 7 years of age by interviewing caregivers. Maternal employment status (outcome) was collected at the 7- and 10-year follow-up waves. Robust Poisson regression models were used to estimate associations.

**Results:**

After adjustment for maternal and household characteristics, women were more likely to be unemployed at child age 10 if the child had, up to age 7, any of the following suspected problems: an autism spectrum disorder (PR = 1.73; 95% CI 1.07, 2.79), developmental delay (PR = 1.58; 95% CI 1.20, 2.06), externalising behaviours (PR = 1.29; 95% CI 1.11, 1.50) or learning problems (PR = 1.26; 95% CI 1.07, 1.48). When the exposure was restricted to clinically diagnosed disorders, the magnitude of associations remained similar but estimates were less precise. Associations with unemployment were stronger at child age 10 (prospective analyses), than at child age 7 (cross-sectional).

**Conclusion:**

Having a child with learning, developmental or behavioural problems, or an autism spectrum disorder up to age 7 was associated with maternal unemployment three years later, even in a less affluent European economy where the dual-earner family structure is often necessary to make ends meet.

**Supplementary Information:**

The online version contains supplementary material available at 10.1007/s00127-023-02464-6.

## Introduction

The participation of women in the workforce has increased substantially throughout the last decades and, in European Union (EU) countries, the share of employed women is among the highest in the world. Still, the gender gap in work participation, although decreasing, remains considerable [1], and it increases with the number of children in the household [2]. In 2021, women with children were less likely to be in employment compared to women without children (72.4% vs. 77.2%), while men showed the opposite pattern (90.0% vs. 80.9%) [3]. Mothers commonly adjust their work participation to meet family needs [4], and make employment decisions based on a number of family-related factors, including their children’s health and behaviour [5]. Since women are traditionally the primary caregiver, maternal work participation decreases around birth (67.0% when youngest child is 0–5 years of age) and increases as the child ages (75.0% when child is 6–11 years and 77.8% when child is over 11 years), thus resembling employment of women with no children [3, 6]. However, this return-to-work pattern is not evident among mothers of children with special needs [7].

The impact of children’s health on maternal work participation is likely to vary across countries with varying occupational gender gaps, as well as diverse social and economic contexts. While the employment rate of mothers with children aged 0–14 years in the EU was 68.2% in 2014, Portugal stood out with a proportion of maternal employment over 75%, together with Nordic countries such as Denmark and Sweden, and more distant from the other Southern European countries such as Greece, Italy, and Spain, where maternal employment was below 60% [8]. In addition, and according to the Organisation for Economic Co-operation and Development (OECD), Portugal is one of the few countries where over 90% of employed mothers work full-time hours (over 30–35 h weekly), along with the Czech Republic, Latvia, Poland and Slovenia [8]. Thus, Portugal combines high rates of maternal full-time employment with less developed state-supported care services when compared to other countries, such as the Scandinavian countries, making grandparents important childcare providers [9]. It is also characterised by labour market factors that generally increase female employment [1] such as low wages and lack of availability of part-time jobs [9].

Previous studies in the more affluent countries have found an association between maternal employment-related outcomes (e.g. unemployment, non-employment, maternal/family employment trajectories) and having a child with developmental or behavioural problems. These include children with language impairment [10], developmental disabilities [5], internalising/externalising behavioural problems [11], as well as children with mental health care needs overall, or with less prevalent conditions such as autism [5]. In addition to the direct impact of child neurodevelopmental and behavioural problems on the family, maternal nonparticipation in the workforce may indirectly add to the long-term impact of these conditions, namely on family well-being. However, there is scarce evidence on the impact of children’s poor mental health on maternal employment in less affluent European economies where the dual-earner family structure is often necessary to make ends meet—although Portugal shares a relatively high maternal employment, it differs from countries included in previous research through its lower wages, less availability of part-time jobs, and different cultural and childcare services characteristics [8, 9, 12, 13]. Thus, we aimed to estimate the association between children suspected or diagnosed neurodevelopmental or behavioural problems up to age seven and maternal unemployment at child age 7 and 10 in a Portuguese birth cohort. We hypothesise that (a) mothers of children with neurodevelopmental or behavioural problems are more likely to be unemployed, with a lasting effect up to late childhood years, even in a country with traditionally high maternal workforce participation, and that (b) this is true even in households with a lower socioeconomic position, where maternal unemployment is less likely to be an individual choice.

## Methods

### Participants

This study was embedded in Generation XXI, a prospective population-based birth cohort study, which has been described in detail elsewhere [14, 15]. Newborns and their mothers were recruited up to 72 h after birth between April 2005 and August 2006 at the five public maternity wards providing maternal and newborn care and covering the metropolitan area of Porto, which included 91.6% of all deliveries in the catchment area at the time of recruitment. Of the invited mothers, 91.4% accepted to participate and a total of 8495 mothers and their 8647 children were enrolled at baseline. The cohort has been followed-up regularly, with five assessment waves completed, at birth, 4, 7, 10 and 13 years of age. This study was based on data from the 7- and 10-year follow-up waves (from April 2012 to April 2014 and July 2015 to July 2017, respectively).

**Fig. 1:**
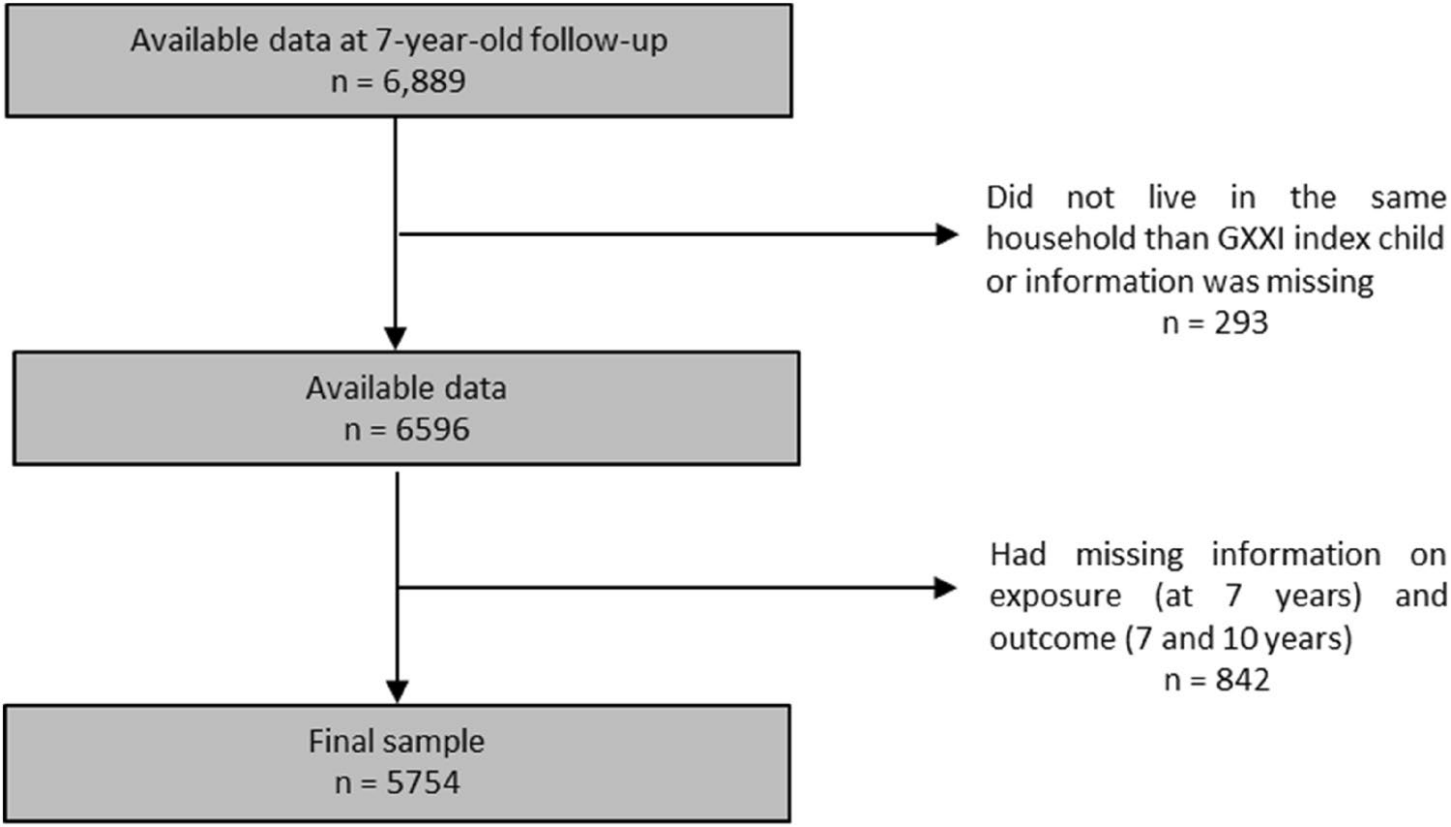
Flow diagram of final sample selection

For the purpose of this study, we included 5754 mothers and their children, that lived in the same household and had complete information on child neurodevelopmental and behavioural problems at 7 years of age, and on maternal employment at child age 7 and 10 (Fig. 1). When compared to the remaining cohort participants (*n* = 2741), mothers included in the analysis were slightly older (29.8 vs. 27.3 years), and more likely to have a higher socioeconomic position—higher educational level (11.0 vs. 9.2 schooling years) and monthly household income with more than EUR 2000 (17.4% vs. 11.8%)—than mothers not included in the analysis.

The Generation XXI cohort study protocol was approved by the Ethics Committee of São João Hospital/University of Porto Medical School and complies with the Ethical Principles expressed in the Helsinki Declaration and with the current national legislation. The project was registered with the Portuguese Authority for Data Protection. Written informed consent was obtained at all assessments.

### Data collection

Child neurodevelopmental and behavioural problems (exposure) were assessed when the child was 7 years of age (only measured at the 7-year assessment wave), as well as covariates. Maternal unemployment (outcome) was measured on both the 7- and the 10-year follow-ups.

#### Child neurodevelopmental and behavioural problems

When the child was 7 years of age, caregivers reported whether they or their child’s teacher had ever suspected that the child had (a) learning, (b) attention, (c) language, (d) behavioural or (e) socialisation problems, (f) developmental delay or (g) an autism spectrum disorder (including Asperger’s syndrome). They were also asked whether the children had a clinical diagnosis of any of these problems. The question on behavioural problems was open-ended and the answers were classified in ‘externalising behaviours’ or ‘other problems’, both suspected and diagnosed, separately. Externalising behaviours included impulse-control disorders, hyperactivity disorders and aggressive behaviour. The ‘other problems’ category included socialisation problems, anxiety, depression, fear, lack of self-confidence and shyness. The number of both suspected and diagnosed problems were separately categorised into ‘no problems’, ‘one problem’ and ‘two or more problems’.

#### Maternal unemployment

When the child was 7 years of age, mothers were asked about their employment status, which was later dichotomized into ‘unemployed’ vs. the remaining categories (full- or part-time worker, unpaid family worker, student, retired, homemaker, or other situation). At the 10-year assessment wave, mothers were asked whether they were currently unemployed, and whether they had been unemployed since 2009 (corresponding to child age 4) as well as the duration of the longest unemployment spell, in months. The latter was categorised into ‘not unemployed’, ‘≤ 12 months’, ‘13–36 months’ and ‘> 36 months’.

#### Covariates

A number of variables that may influence the association between child neurodevelopmental and behavioural problems and maternal work participation were included in the study. Maternal age and education at the 7-year follow-up were retrieved, since it has been observed that the lower the level of education, the more affected the employment rate is by the presence of children in the household [16]. Education was recorded as the number of completed schooling years and grouped as ≤ 9, 10–12, or > 12 years of education.

Data on household characteristics were also taken into account due to the impact of time spent in household-related tasks on paid work [7]. Single mother families were defined as women living with the index child but without the biological father or another partner. Data on having a singleton/multiple index pregnancy and on having other children and/or stepchildren below age 6 were also retrieved.

Mother-reported history of diagnosed mental disorders was also included as an adjustment variable, due to the association between maternal psychopathology and both presence and report on child’s behavioural and emotional problems (particularly depression and anxiety) [17, 18], as well as to its link to decreased employment [19, 20]. Maternal mental disorders were computed based on two binary questions (diagnosis of depression that requires regular medical care and previous diagnosis of postpartum depression) and an open-ended one on other health problems, which included mental disorders. Women were considered as having a diagnosis of a mental disorder if they had at least one positive response in any of these three variables. All adjustment variables were retrieved from the 7-year follow-up.

### Data analysis

**Fig. 2:**
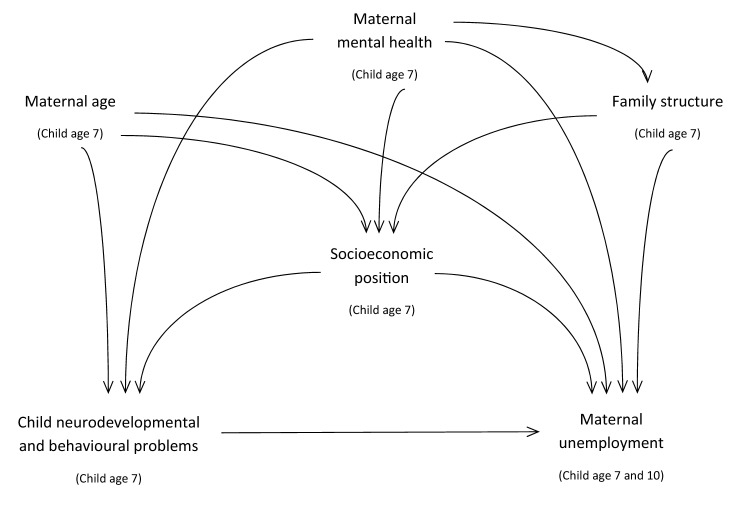
Causal diagram displaying the association between child neurodevelopmental or behavioural problems and maternal unemployment, and potential confounders, with time of assessment

Several factors may affect both child’s neurodevelopment and behaviour, and maternal work participation, thus potentially confounding our effect estimates (Fig. 2), thus, we adopted a 3-step approach. Robust Poisson regression models were used to calculate prevalence ratios (PRs) and 95% confidence intervals (CIs) to estimate associations between child neurodevelopmental or behavioural problems up to 7 years of age (suspected and diagnosed, separately) and maternal unemployment when the child was 7 and 10 years old (model 1). The analysis was further adjusted for maternal age and educational level (model 2). The fully adjusted model was additionally adjusted for family structure-related variables, including being part of a single mother household, having a singleton/multiple pregnancy and having other children under the age of 6 years in the household, as well as for maternal history of diagnosed mental disorders (model 3). The fully adjusted model was also fitted using maternal unemployment at both 7- and 10-year assessment waves as outcome (vs. all other situations). Ordinal logistic regression was used to calculate odds ratios (ORs) in order to estimate the association between child neurodevelopmental or behavioural problems until 7 years of age and duration of maximum maternal unemployment spell between child’s age 4 and 10 years. Statistical analysis was performed using the statistical software IBM SPSS Statistics for Windows, Version 21.0 (Chicago, IL, USA).

### Sensitivity analysis

Assuming that unemployment may be dependent on the affluence of the household, estimates were computed after excluding families of the highest household income category (more than EUR 3000; *n* = 501). Additional sensitivity analyses to assess the impact of long-term unemployment not attributable to child-related factors were carried out by excluding mothers who were unemployed at child birth (*n* = 995). To exclude the possible role of child organic diseases on maternal unemployment, estimates were also computed after excluding mothers whose children had a diagnosis of cerebral palsy, congenital malformations, growth problems, heart problems, renal problems, liver problems, epilepsy, type 1 diabetes, or lymphoma/leukaemia (*n* = 1287).

## Results

In our study, the most frequently suspected neurodevelopmental and behavioural problems in children during the first 7 years of life were attention problems (21.0%) followed by language problems (16.0%). The latter was also the most prevalent clinically diagnosed problem (11.4%). Autism spectrum disorders had the lowest prevalence, both suspected (0.9%) and diagnosed (0.5%). Table 1 summarises maternal unemployment at child age 7 and 10, according to maternal individual and household-related characteristics. When the child was 7 years of age, most mothers in our sample had more than 30 years of age (88.4%), had 12 or less years of schooling (69.9%), lived with a partner (86.0%), and had no children under the age of 6 in the household (75.0%). Having a mental disorder diagnosis was reported by 10.4% of the mothers. The prevalence of maternal unemployment was 18.5% at the 7-year follow-up, 16.7% when the child was 10 years of age, and 8.7% at both assessment waves. Unemployment was more frequent among women under 30 years of age, women with 9 or less schooling years, women living in single mother households, and with a history of a mental disorder diagnosis.

**Table 1:** Prevalence of unemployment among mothers at child age 7 and 10 by maternal characteristics, in Generation XXI

	Total	Unemployed at 7	Unemployed at 10	Unemployed at 7 and 10
	*n* = 5754 *n* (%)	*n* = 1065 *n* (%)	*n* = 963 *n* (%)	*n* = 499 *n* (%)
Maternal characteristics				
Age (years)				
≤ 30	665 (11.6)	191 (28.7)	156 (23.5)	78 (11.7)
31–40	3612 (62.8)	611 (16.9)	543 (15.0)	279 (7.7)
> 40	1474 (25.6)	263 (17.8)	263 (17.8)	142 (9.6)
Education (schooling years)				
≤ 9	2274 (39.6)	603 (26.5)	565 (24.8)	311 (13.7)
10–12	1743 (30.3)	313 (18.0)	252 (14.5)	134 (7.7)
> 12	1729 (30.1)	147 (8.5)	145 (8.4)	53 (3.1)
Single mother family				
No	4939 (86.0)	883 (17.9)	798 (16.2)	408 (8.3)
Yes	805 (14.0)	181 (22.5)	162 (20.1)	90 (11.2)
Singleton/multiple pregnancy				
Singleton	5647 (98.1)	1043 (18.5)	949 (16.8)	493 (8.7)
Multiple	107 (1.9)	22 (20.6)	14 (13.1)	6 (5.6)
Children below age 6				
No	4293 (75.0)	784 (18.3)	717 (16.7)	368 (8.6)
Yes	1434 (25.0)	276 (19.2)	240 (16.7)	128 (8.9)
Maternal mental disorders				
No	5153 (89.6)	856 (17.8)	760 (15.8)	395 (8.2)
Yes	601 (10.4)	209 (22.4)	203 (21.7)	104 (11.1)

Associations between child neurodevelopmental or behavioural problems and maternal unemployment at child age 7 and 10 are presented in Table 2. Women had higher risk of unemployment at the 7-year follow-up if their child had suspected developmental delay (PR_crude_ = 1.60; 95% CI 1.22, 2.09), learning problems (PR_crude_ = 1.53; 95% CI 1.32, 1.77) or externalising behaviours (PR_crude_ = 1.31; 95% CI 1.13, 1.52). A similar pattern was found for clinically diagnosed problems, with developmental delay (PR_crude_ = 1.69; 95% CI 1.26, 2.28), learning problems (PR_crude_ = 1.56; 95% CI 1.26, 1.93) and externalising behaviours (PR_crude_ = 1.29; 95% CI 0.99, 1.67) showing an association with increased maternal unemployment at child age 7. After adjustment for maternal age and educational level (model 2), most estimates were attenuated but the same overall pattern remained, with positive associations for suspected developmental delay (PR_adj_ = 1.36; 95% CI 1.04, 1.77), learning problems (PR_adj_ = 1.25; 95% CI 1.08, 1.44) or externalising behaviours (PR_adj_ = 1.15; 95% CI 0.99, 1.33). Regarding diagnosed problems, only developmental delay (PR_adj_ = 1.42; 95% CI 1.06, 1.91) and learning problems (PR_adj_ = 1.31; 95% CI 1.06, 1.61) showed an association with maternal unemployment at child age 7 (model 2). Additional adjustment for other reasons for a demanding home situation and maternal mental health (model 3) was responsible for further attenuation of estimates, and associations remained strongest for developmental delay and learning problems, both suspected (PR_adj_ = 1.33; 95% CI 1.02, 1.74 and PR_adj_ = 1.20; 95% CI 1.04, 1.40, respectively) and diagnosed (PR_adj_ = 1.39; 95% CI 1.04, 1.87 and PR_adj_ = 1.28; 95% CI 1.04, 1.57, respectively).

**Table 2:** Associations between child neurodevelopmental or behavioural problems and maternal unemployment at child age 7 and 10 (prevalence ratio, PR, and respective 95% confidence interval, CI)

		Model 1		Model 2		Model 3		Model 3
	*N* (%) (*n* = 5754)	Child age 7	Child age 10	Child age 7	Child age 10	Child age 7	Child age 10	7 and 10
Suspected								
Learning problems								
No	5184 (90.2)	1	1	1	1	1	1	1
Yes	562 (9.8)	1.53 (1.32–1.77)	1.56 (1.33–1.83)	1.25 (1.08–1.44)	1.27 (1.08–1.49)	1.20 (1.04–1.40)	1.26 (1.07–1.48)	1.29 (1.03–1.63)
Attention problems								
No	4528 (79.0)	1	1	1	1	1	1	1
Yes	1207 (21.0)	1.12 (0.99–1.28)	1.03 (0.90–1.19)	1.04 (0.92–1.18)	0.97 (0.84–1.11)	1.02 (0.90–1.16)	0.97 (0.84–1.11)	1.08 (0.89–1.31)
Language problems								
No	4825 (84.0)	1	1	1	1	1	1	1
Yes	918 (16.0)	1.14 (0.99–1.32)	1.17 (1.01–1.36)	1.06 (0.93–1.22)	1.09 (0.94–1.25)	1.07 (0.93–1.22)	1.09 (0.94–1.27)	1.08 (0.87–1.34)
Externalising behaviours								
No	5080 (88.7)	1	1	1	1	1	1	1
Yes	649 (11.3)	1.31 (1.13–1.52)	1.50 (1.29–1.74)	1.15 (0.99–1.33)	1.32 (1.14–1.53)	1.11 (0.96–1.29)	1.29 (1.11–1.50)	1.41 (1.13–1.74)
Developmental delay								
No	5596 (97.7)	1	1	1	1	1	1	1
Yes	133 (2.3)	1.60 (1.22–2.09)	1.92 (1.49–2.49)	1.36 (1.04–1.77)	1.61 (1.24–2.11)	1.33 (1.02–1.74)	1.58 (1.20–2.06)	1.54 (1.04–2.26)
Autism								
No	5670 (99.1)	1	1	1	1	1	1	1
Yes	54 (0.9)	1.10 (0.65–1.87)	1.55 (0.99–2.45)	1.25 (0.74–2.12)	1.77 (1.10–2.85)	1.20 (0.71–2.05)	1.73 (1.07–2.79)	1.42 (0.65–3.08)
Other problems^a^								
No	5387 (93.9)	1	1	1	1	1	1	1
Yes	352 (6.1)	1.08 (0.87–1.34)	1.11 (0.88–1.39)	1.06 (0.85–1.31)	1.09 (0.87–1.37)	1.04 (0.83–1.29)	1.07 (0.85–1.35)	1.07 (0.77–1.49)
Diagnosed								
Learning problems								
No	5510 (96.0)	1	1	1	1	1	1	1
Yes	231 (4.0)	1.56 (1.26–1.93)	1.51 (1.20–1.91)	1.31 (1.06–1.61)	1.26 (1.00–1.59)	1.28 (1.04–1.57)	1.22 (0.96–1.54)	1.31 (0.95–1.82)
Attention problems								
No	5438 (95.0)	1	1	1	1	1	1	1
Yes	288 (5.0)	1.13 (0.90–1.43)	1.31 (1.04–1.64)	1.03 (0.82–1.28)	1.19 (0.95–1.49)	1.01 (0.80–1.26)	1.15 (0.91–1.44)	1.26 (0.92–1.72)
Language problems								
No	5078 (88.6)	1	1	1	1	1	1	1
Yes	653 (11.4)	1.03 (0.87–1.22)	1.16 (0.98–1.37)	0.97 (0.82–1.14)	1.08 (0.91–1.27)	0.97 (0.82–1.14)	1.06 (0.90–1.26)	0.94 (0.72–1.22)
Externalising behaviours								
No	5521 (96.7)	1	1	1	1	1	1	1
Yes	191 (3.3)	1.29 (0.99–1.67)	1.49 (1.16–1.93)	1.11 (0.86–1.44)	1.31 (1.03–1.67)	1.07 (0.83–1.38)	1.23 (0.96–1.59)	1.44 (1.02–2.04)
Developmental delay								
No	5626 (98.3)	1	1	1	1	1	1	1
Yes	100 (1.7)	1.69 (1.26–2.28)	1.82 (1.34–2.47)	1.42 (1.06–1.91)	1.50 (1.10–2.06)	1.39 (1.04–1.87)	1.42 (1.03–1.96)	1.54 (1.00–2.39)
Autism								
No	5692 (99.5)	1	1	1	1	1	1	1
Yes	30 (0.5)	0.72 (0.29–1.79)	1.60 (0.88–2.90)	0.78 (0.31–1.96)	1.73 (0.94–3.18)	0.75 (0.30–1.89)	1.66 (0.88–3.12)	1.20 (0.40–3.60)
Other problems^a^								
No	5636 (98.3)	1	1	1	1	1	1	1
Yes	98 (1.7)	1.11 (0.74–1.64)	1.29 (0.88–1.89)	1.15 (0.78–1.71)	1.36 (0.93–2.00)	1.11 (0.75–1.65)	1.25 (0.83–1.87)	1.21 (0.67–2.20)
Nr. of suspected problems								
0	3514 (62.1)	1	1	1	1	1	1	1
1	1219 (21.5)	1.13 (0.99–1.29)	1.09 (0.94–1.26)	1.07 (0.94–1.22)	1.04 (0.90–1.20)	1.08 (0.94–1.23)	1.05 (0.91–1.21)	1.10 (0.89–1.36)
2+	924 (16.3)	1.36 (1.19–1.56)	1.36 (1.18–1.57)	1.16 (1.01–1.33)	1.17 (1.01–1.35)	1.13 (0.99–1.30)	1.14 (0.98–1.32)	1.28 (1.04–1.57)
Nr. of diagnosed problems								
0	4748 (84.5)	1	1	1	1	1	1	1
1	552 (9.8)	0.91 (0.75–1.10)	1.08 (0.89–1.31)	1.04 (0.96–1.13)	1.04 (0.86–1.25)	0.87 (0.72–1.05)	1.03 (0.86–1.25)	0.76 (0.55–1.04)
2+	321 (5.7)	1.32 (1.08–1.62)	1.42 (1.15–1.75)	1.16 (1.05–1.27)	1.23 (1.00–1.51)	1.11 (0.91–1.35)	1.18 (0.95–1.46)	1.32 (0.99–1.76)

Model 1—crude associations

Model 2—adjusted for maternal age and educational level, in years

Model 3—adjusted for maternal age (in years), maternal educational level (in years), single mother household, singleton/multiple pregnancy, having children with less than 6 years of age in household and maternal history of a diagnosed mental disorder

^a^Includes socialisation problems, anxiety problems, depression, fear, lack of self-confidence and shyness

Associations were overall stronger with unemployment at child age 10, when compared to child age 7. Women were more likely to be unemployed at child age 10 if their child was suspected to have an autism spectrum disorder (PR_adj_ = 1.73; 95% CI 1.07, 2.79), developmental delay (PR_adj_ = 1.58; 95% CI 1.20, 2.06), externalising behaviours (PR_adj_ = 1.29; 95% CI 1.11, 1.50) or learning problems (PR_adj_ = 1.26; 95% CI 1.07, 1.48) (model 3). When the exposure was restricted to clinically diagnosed disorders, the magnitude of associations remained similar to suspected disorders but estimates were less precise, and only seemingly more severe problems, such as developmental delay (PR_adj_ = 1.42; 95% CI 1.03, 1.96), showed a clear association with maternal unemployment (model 3).

Estimates for unemployment at both waves of assessment were overall higher for mothers who had children with suspected and diagnosed developmental delay, externalising behaviours and learning problems (Table 2). There were no clear associations between attention or language problems, suspected or diagnosed, and maternal unemployment at any assessment wave after adjustment for covariates. However, there was an association between having children with two or more neurodevelopmental or behavioural problems, particularly suspected ones, and maternal unemployment (PR_adj_ = 1.13; 95% CI 0.99, 1.30 and PR_adj_ = 1.14; 95% CI 0.98, 1.32, for child age 7 and 10, respectively) (model 3).

When looking into the longest unemployment spell, women were more likely to have unemployment spells of more than 3 years between child age 4 and 10, if the child had suspected or diagnosed developmental delay (OR_adj_ = 2.06; 95% CI 1.48, 2.87 and OR_adj_ = 1.93; 95% CI 1.32, 2.83, respectively), or learning problems (OR_adj_ = 1.31; 95% CI 1.11, 1.56 and OR_adj_ = 1.33; 95% CI 1.03, 1.73, respectively), or a suspected autism spectrum disorder (OR_adj_ = 1.68; 95% CI 0.98, 2.86) (results not shown in tables).

**Fig. 3:**
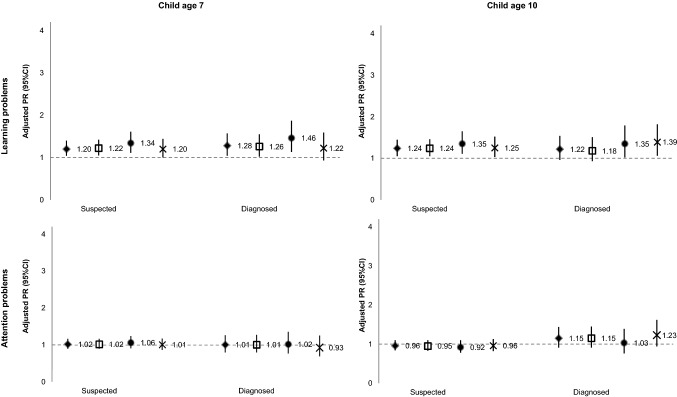

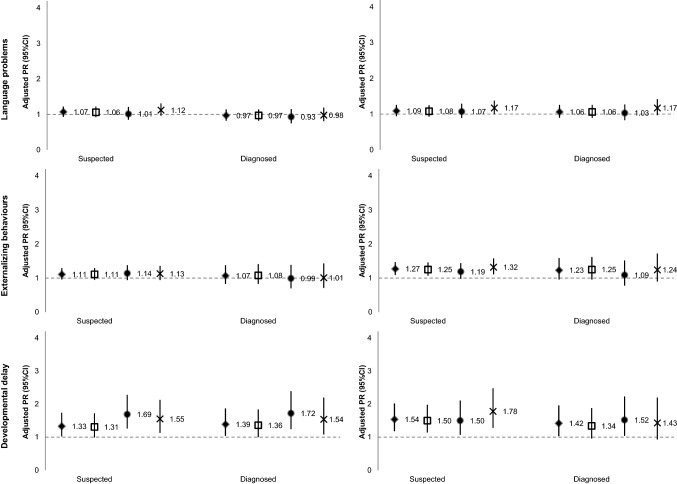

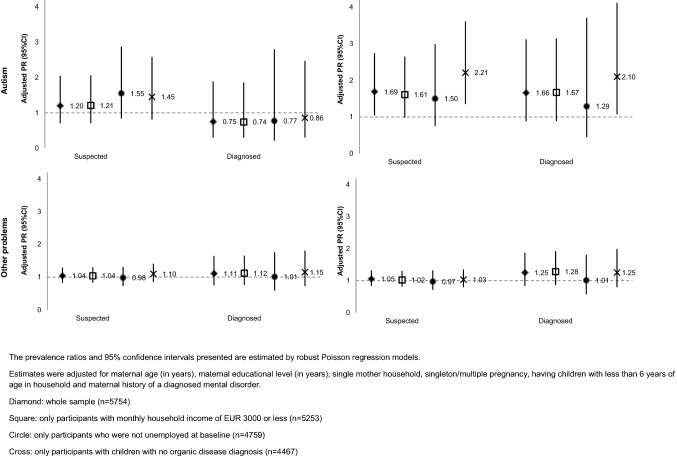
Sensitivity analysis: adjusted prevalence ratios (95% CI) for associations between child neurodevelopmental or behavioural problems and maternal unemployment at child age 7 and 10

Figure 3 shows the results of the sensitivity analyses excluding households with a monthly household income over EUR 3000, mothers who were unemployed at baseline, or mothers whose children had an organic disease diagnosis (estimates presented in supplemental Tables 1, 2 and 3). When excluding families with a household income of more than EUR 3000, estimates were similar to those found for the whole sample. When excluding mothers unemployed at child birth, results showed an overall increase in the likelihood of maternal unemployment, particularly at the 7-year follow-up, when children had learning problems (PR_suspected_ = 1.34; 95% CI 1.11, 1.61 and PR_diagnosed_ = 1.46; 95% CI 1.13, 1.87) or developmental delay (PR_suspected_ = 1.69; 95% CI 1.26, 2.28 and PR_diagnosed_ = 1.72; 95% CI 1.24, 2.39). After excluding mothers whose children had an organic disease diagnosis, maternal unemployment was more likely, at the 7-year assessment, when children had a developmental delay (PR_suspected_ = 1.55; 95% CI 1.13, 2.13 and PR_diagnosed_ = 1.54; 95% CI 1.08, 2.20) and, at the 10-year assessment, when children had an autism spectrum disorder (PR_suspected_ = 2.21; 95% CI 1.35, 3.61 and PR_diagnosed_ = 2.10; 95% CI 1.07, 4.12) and a suspected developmental delay (PR_suspected_ = 1.78; 95% CI 1.28, 2.48), but estimates were less precise.

## Discussion

### Main findings

In our study, mothers of children with learning problems, externalising behaviours, developmental delay or an autism spectrum disorder up to age seven had a higher likelihood of being unemployed at child age 10. Regarding learning problems, developmental delay and autism, these findings held among previously employed mothers, and associations were stronger among families with a low- to middle-category household income and mothers of children without chronic organic diseases. Previous research has shown that families of children with mental health care needs are more likely to cut work hours or to stop work altogether when compared to families with children without mental health care needs or with children with other health care needs [21, 22]. Moreover, although maternal work participation typically increases as the child grows [6], this return-to-work pattern is not apparent among mothers of children with special health care needs [7]. This lasting effect on maternal work participation is reflected by our findings where nearly all estimates increased between the 7- and 10-year follow-ups, thus suggesting that children’s behavioural and developmental problems may have a sustained long-term impact on maternal employment, in addition to their direct impact on overall family well-being. This study adds to our understanding of the impact of a child’s neurodevelopmental or behavioural problems on maternal unemployment by using a large prospective population-based birth cohort, and particularly by focusing on school-age children of a less affluent economy, such as the southern European one, where research on the subject is scarce.

### Interpretation of findings

Although evidence concerning learning disabilities’ impact on maternal work participation is scarce, previous research has shown an association between children’s intellectual disability and maternal work participation [23, 24], which may include learning problems, among other types of disorders. Concerning behaviour problems, our results are in line with findings from previous studies in toddlers and pre-schoolers to be associated with lower maternal employment [10, 11]. Behaviour problems in early adolescents have also been shown to contribute to the multiple barriers to women’s accessing and retaining stable and quality employment, particularly in economically disadvantaged families [25]. Although our assessment of behaviour problems may entail a myriad of different emotional and developmental problems of varying severity, previous research comparing the impact on families of having young adults with intellectual disabilities (namely Down syndrome, cerebral palsy and autism) showed that the relationship between diagnostic group and maternal well‐being was almost entirely accounted for by the level of behaviour problems [26]. Regarding our findings that having a child with a developmental delay is a predictor of maternal unemployment with lasting effects up until middle childhood, previous research has found that mothers with children with developmental disabilities had lower rates of overall employment and greater likelihood of part-time employment even as their children grew older [27].

Despite its low prevalence in our sample, we also found an association between autism spectrum disorders and maternal unemployment. Autism spectrum disorders have also been found to preclude maternal employment and to decrease the number of hours worked per week [28–30]. In fact, children with an autism spectrum disorder often have co-occurring conditions that require a broader range of health services, thus creating additional family burden, whether it is financial, stress or other mental problems for families [5, 26, 31, 32].

Although we found no association with other, seemingly less severe, neurodevelopmental or behavioural problems, previous findings showed that mothers with a preschool child with language impairment had increased risk of not being employed and of taking long-term sick leaves [10]. Since our outcome, unemployment, consists of an extreme measure of maternal nonparticipation at work, we expect that only the more severe and chronic child conditions, i.e. the ones that require frequent health services utilisation or cause serious functional disability such as developmental delay, learning problems and autism spectrum disorders, will have an effect on maternal unemployment. These findings held when looking into the longest unemployment spell since child age 4 as an outcome, where women were more likely to have unemployment spells of more than 3 years if the child had either suspected or diagnosed developmental delay or learning problems, or a suspected autism spectrum disorder.

Children’s health status may constitute an important risk factor for maternal unemployment, affecting thus a family’s work-life balance. Most research on child healthcare needs and maternal work participation focuses on preschool children. Although access to high-quality childcare for toddlers and pre-schoolers is a major factor for parents’ employment decisions, childcare for school-age children must also be considered [33]. In fact, in about half of the European Union Member States, being a mother decreases the likelihood of working 40 or more hours per week; whereas Portugal stands out as one of the few European countries where mothers are more likely to work more than 40 h per week than childless women [34]. The latter might be explained by cultural differences, less flexible working arrangements, long working hours and the perception that leadership cannot be executed part-time [35]. In addition to schedule differences between child and parents affecting all mothers and families, school-age children may inhibit maternal employment via other possible explanations, particularly if the child has special care needs.

Decreased maternal work participation may be the product of an individual and voluntary choice or the consequence of a suboptimal performance at home or at work. The conflict between job and parenting demands may be higher for parents of children with emotional or behavioural problems, leading to the disruption of work-family balance and increased levels of stress [33]. Stress might lead parents, and mothers in particular, to decrease the number of hours worked or even to stop work altogether. Decreased maternal work participation may also be explained by the fact that childcare activities could interfere with job performance and work hours, since children may require enhanced attention and supervision, meetings with educational personnel, and medical visits [25, 36]. The increased need to provide childcare may interfere with mothers’ ability to not only perform other personal and family-related activities, but also with the ability to meet occupational requirements and to retain employment. Therefore, having a child with special care needs is likely to increase maternal disadvantage regarding career advancement, income opportunities and pension entitlements, which will economically affect families in the long term, leaving mothers and their families more vulnerable to adversity. There is even an intergenerational transmission of unemployment [37]. Also, leaving employment for a long period of time may have negative consequences on mothers since, depending on the child’s condition, work may have a respite effect on mothers of older children with special needs [38].

Our findings are particularly relevant and must be understood in view of the Portuguese context, a less affluent European economy, with relatively high maternal workforce participation, and where reconciling work and family via part-time jobs is rarely an option [12, 34]—in our sample, only 7.5% of mothers worked part-time at child age 7. In Portugal, parents with children under 12 years of age (no age limit in the case of a child with an illness or disability living in the same household) are entitled to work part-time or to flexible working arrangements, which means that the employee may choose, within certain limits, when to start and finish daily work without reducing working hours [39]. However, these entitlements have a limited impact on the Portuguese labour market—and flexibility is higher in the more qualified occupations [13]. In Portugal, in 2015, the employment rate of mothers of children under the age of 6 was higher than the employment rate of childless women, followed by Croatia, Slovenia and Luxembourg, while the reverse was observed in most European countries [40]. Furthermore, female employment rates remain fairly stable as the age of the youngest child increases [6]. Also, female employment rates increase in the presence of one or two children when compared to childless women, which is consistent with the cost and the ‘logistics’ involved in childcare; a sharper drop is only observed from the third child onwards [6].

### Strengths and limitations

Although previous studies have looked into the association between child neurodevelopmental or behavioural problems and maternal work participation, prospective research in school-age children of a less affluent economy, such as the southern European one, with its cultural and childcare services characteristics, is scarce. However, the interpretation of our findings needs to take into account several methodological issues. First, due to the nature of the data collected, we were not able to explore the severity of the child’s condition or the specific types of problems within the larger groups used to assess exposure, which may have underestimated our results on some of the conditions studied. Second, we only had information on the health of the child that was part of the Generation XXI birth cohort, and so the impact of other potential siblings’ health problems on maternal employment could not be measured. Third, we did not include partner’s work participation, which may influence maternal employment, due to missing information; yet, including the variable on monthly household income in the sensitivity analysis may partially account for it. Also, differential losses to follow-up, particularly regarding socioeconomic position, should be taken into account when interpreting our findings. However, the magnitude of those differences was generally small, suggesting that our association estimates were not largely biased due to selective losses to follow-up, as previous longitudinal research on mothers has shown [41]. Also, sensitivity analyses excluding mothers with higher educational level (data not shown) and more affluent households did not change the main conclusions of our study, although some estimates were slightly attenuated, supporting the fact that our results were not greatly biased by losses to follow-up. Lastly, although our cohort’s participants reported on the child’s diagnosis of a neurodevelopmental or behavioural problem, and we relied on self-reported information without clinical validation, it may have introduced some level of misclassification. Thus, inferences must be made with caution.

## Conclusion

The present study showed that having a child with a learning, developmental or behavioural problem, or an autism spectrum disorder was associated with maternal unemployment up to age 10, even in a less affluent European economy where the dual-earner family structure is often necessary to make ends meet. Children’s learning, developmental and behavioural problems, and autism spectrum disorders, thus, entail long-term consequences related to families’ well-being, financial condition, and companies’ human capital. A more comprehensive depiction of the employment patterns of mothers, including flexible work arrangements, needs to be addressed and developed, in order for mothers, families and companies to prosper. Research that monitors the implementation of existing family-friendly work policies, namely the right to request flexible work arrangements until child is 12 years old and telework for parents with children below 3 years of age, will be needed to ensure successful outcomes. Moreover, access to childcare needs to be improved and research needs to examine how services specifically for children with disabilities are delivered.

### Supplementary Information

Below is the link to the electronic supplementary material.

Supplementary file1 (DOCX 68 KB)

## Data Availability

The data from Generation XXI are not publicly available due to privacy or ethical restrictions. The data can be made available for research proposals on request to the Generation XXI Executive Committee (generationxxi@ispup.up.pt).
