# Supplementary material for: Association of child neurodevelopmental or behavioural problems with maternal unemployment in a population-based birth cohort
Source: Soc Psychiatry Psychiatr Epidemiol. 2023 Mar 26;59(4):643–55. doi: 10.1007/s00127-023-02464-6 (PMC10960748; doi:10.1007/s00127-023-02464-6)
Supplement: Supplementary file 1 — Supplementary file1 (DOCX 68 KB) [file 127_2023_2464_MOESM1_ESM.docx]

**Supplemental table 1.** Associations between child neurodevelopmental or behavioural problems and maternal unemployment at child age 7 and 10 years, after excluding families with household income of more than EUR 3000 (prevalence ratio, PR, and respective 95% confidence interval, CI)

|  |  | **Model 1** | | | **Model 2** | | **Model 3** | | | | **Model 3** |
| --- | --- | --- | --- | --- | --- | --- | --- | --- | --- | --- | --- |
|  | **N (%)**  (n=5253) | **Child age 7** | **Child age 10** | **Child age 7** | | **Child age 10** | | **Child age 7** | **Child age 10** | **7 and 10** | |
| **Suspected** |  |  |  |  | |  | |  |  |  | |
| **Learning problems** |  |  |  |  | |  | |  |  |  | |
| No | 4720 (90.0) | 1 | 1 | 1 | | 1 | | 1 | 1 | 1 | |
| Yes | 525 (10.0) | 1.52 (1.31-1.76) | 1.53 (1.30-1.79) | 1.26 (1.09-1.47) | | 1.26 (1.07-1.49) | | 1.22 (1.05-1.42) | 1.24 (1.05-1.46) | 1.30 (1.03-1.64) | |
| **Attention problems** |  |  |  |  | |  | |  |  |  | |
| No | 4118 (78.6) | 1 | 1 | 1 | | 1 | | 1 | 1 | 1 | |
| Yes | 1118 (21.4) | 1.11 (0.98-1.27) | 1.01 (0.88-1.17) | 1.04 (0.91-1.18) | | 0.96 (0.83-1.10) | | 1.02 (0.90-1.16) | 0.95 (0.83-1.10) | 1.08 (0.88-1.31) | |
| **Language problems** |  |  |  |  | |  | |  |  |  | |
| No | 4395 (83.8) | 1 | 1 | 1 | | 1 | | 1 | 1 | 1 | |
| Yes | 848 (16.2) | 1.14 (0.99-1.31) | 1.16 (0.99-1.34) | 1.06 (0.92-1.22) | | 1.07 (0.93-1.25) | | 1.06 (0.92-1.22) | 1.08 (0.93-1.25) | 1.07 (0.86-1.33) | |
| **Externalizing behaviours** |  |  |  |  | |  | |  |  |  | |
| No | 4633 (88.6) | 1 | 1 | 1 | | 1 | | 1 | 1 | 1 | |
| Yes | 597 (11.4) | 1.28 (1.10-1.50) | 1.45 (1.24-1.70) | 1.14 (0.98-1.33) | | 1.30 (1.12-1.52) | | 1.11 (0.95-1.29) | 1.25 (1.07-1.46) | 1.41 (1.13-1.75) | |
| **Developmental delay** |  |  |  |  | |  | |  |  |  | |
| No | 5106 (97.6) | 1 | 1 | 1 | | 1 | | 1 | 1 | 1 | |
| Yes | 125 (2.4) | 1.54 (1.17-2.03) | 1.83 (1.40-2.39) | 1.33 (1.01-1.75) | | 1.56 (1.19-2.05) | | 1.31 (0.99-1.72) | 1.50 (1.14-1.98) | 1.61 (1.09-2.37) | |
| **Autism** |  |  |  |  | |  | |  |  |  | |
| No | 5172 (99.0) | 1 | 1 | 1 | | 1 | | 1 | 1 | 1 | |
| Yes | 51 (1.0) | 1.11 (0.66-1.88) | 1.47 (0.92-2.36) | 1.26 (0.74-2.13) | | 1.67 (1.02-2.74) | | 1.21 (0.71-2.06) | 1.61 (0.98-2.65) | 1.45 (0.67-3.15) | |
| **Other problems^a^** |  |  |  |  | |  | |  |  |  | |
| No | 4917 (93.8) | 1 | 1 | 1 | | 1 | | 1 | 1 | 1 | |
| Yes | 323 (6.2) | 1.07 (0.86-1.34) | 1.08 (0.85-1.36) | 1.06 (0.85-1.32) | | 1.07 (0.84-1.35) | | 1.04 (0.83-1.30) | 1.02 (0.81-1.30) | 1.10 (0.79-1.54) | |
| **Diagnosed** |  |  |  |  | |  | |  |  |  | |
| **Learning problems** |  |  |  |  | |  | |  |  |  | |
| No | 5023 (95.9) | 1 | 1 | 1 | | 1 | | 1 | 1 | 1 | |
| Yes | 217 (4.1) | 1.51 (1.21-1.87) | 1.44 (1.13-1.83) | 1.28 (1.04-1.59) | | 1.22 (0.96-1.55) | | 1.26 (1.02-1.55) | 1.18 (0.93-1.51) | 1.27 (0.91-1.78) | |
| **Attention problems** |  |  |  |  | |  | |  |  |  | |
| No | 4958 (94.8) | 1 | 1 | 1 | | 1 | | 1 | 1 | 1 | |
| Yes | 270 (5.2) | 1.11 (0.88-1.41) | 1.28 (1.01-1.61) | 1.03 (0.82-1.29) | | 1.19 (0.94-1.49) | | 1.01 (0.80-1.27) | 1.15 (0.91-1.45) | 1.28 (0.93-1.76) | |
| **Language problems** |  |  |  |  | |  | |  |  |  | |
| No | 4633 (88.6) | 1 | 1 | 1 | | 1 | | 1 | 1 | 1 | |
| Yes | 598 (11.4) | 1.03 (0.87-1.23) | 1.15 (0.97-1.37) | 0.97 (0.82-1.14) | | 1.07 (0.90-1.27) | | 0.97 (0.82-1.14) | 1.06 (0.89-1.26) | 0.95 (0.73-1.24) | |
| **Externalizing behaviours** |  |  |  |  | |  | |  |  |  | |
| No | 5037 (96.6) | 1 | 1 | 1 | | 1 | | 1 | 1 | 1 | |
| Yes | 177 (3.4) | 1.26 (0.97-1.65) | 1.45 (1.12-1.89) | 1.12 (0.87-1.46) | | 1.31 (1.02-1.68) | | 1.08 (0.83-1.41) | 1.25 (0.96-1.62) | 1.45 (1.01-2.07) | |
| **Developmental delay** |  |  |  |  | |  | |  |  |  | |
| No | 5134 (98.2) | 1 | 1 | 1 | | 1 | | 1 | 1 | 1 | |
| Yes | 94 (1.8) | 1.60 (1.18-2.18) | 1.67 (1.21-2.31) | 1.38 (1.02-1.87) | | 1.42 (1.02-1.97) | | 1.36 (1.00-1.84) | 1.34 (0.96-1.88) | 1.62 (1.05-2.51) | |
| **Autism** |  |  |  |  | |  | |  |  |  | |
| No | 5192 (99.4) | 1 | 1 | 1 | | 1 | | 1 | 1 | 1 | |
| Yes | 29 (0.6) | 0.71 (0.29-1.76) | 1.59 (0.88-2.88) | 0.77 (0.31-1.93) | | 1.73 (0.94-3.18) | | 0.74 (0.30-1.86) | 1.67 (0.89-3.14) | 1.21 (0.40-3.62) | |
| **Other problems^a^** |  |  |  |  | |  | |  |  |  | |
| No | 5142 (98.2) | 1 | 1 | 1 | | 1 | | 1 | 1 | 1 | |
| Yes | 92 (1.8) | 1.12 (0.76-1.66) | 1.32 (0.90-1.94) | 1.16 (0.79-1.72) | | 1.39 (0.95-2.03) | | 1.12 (0.76-1.66) | 1.28 (0.86-1.92) | 1.23 (0.68-2.24) | |
| **Nr. of suspected problems** |  |  |  |  | |  | |  |  |  | |
| 0 | 3199 (62.0) | 1 | 1 | 1 | | 1 | | 1 | 1 | 1 | |
| 1 | 1097 (21.2) | 1.11 (0.96-1.27) | 1.07 (0.92-1.24) | 1.06 (0.92-1.21) | | 1.03 (0.89-1.19) | | 1.06 (0.93-1.22) | 1.04 (0.89-1.20) | 1.10 (0.89-1.37) | |
| 2+ | 867 (16.8) | 1.34 (1.17-1.54) | 1.31 (1.13-1.52) | 1.16 (1.01-1.33) | | 1.15 (0.99-1.33) | | 1.13 (0.98-1.30) | 1.13 (0.97-1.31) | 1.28 (1.04-1.58) | |
| **Nr. of diagnosed problems** |  |  |  |  | |  | |  |  |  | |
| 0 | 4333 (84.5) | 1 | 1 | 1 | | 1 | | 1 | 1 | 1 | |
| 1 | 492 (9.6) | 0.92 (0.75-1.12) | 1.10 (0.90-1.34) | 0.87 (0.72-1.06) | | 1.05 (0.86-1.27) | | 0.87 (0.72-1.06) | 1.05 (0.86-1.27) | 0.79 (0.57-1.09) | |
| 2+ | 303 (5.9) | 1.28 (1.05-1.58) | 1.37 (1.11-1.70) | 1.13 (0.92-1.38) | | 1.21 (0.98-1.49) | | 1.10 (0.90-1.35) | 1.17 (0.94-1.45) | 1.32 (0.98-1.77) | |

Model 1 - crude associations

Model 2 - Adjusted for maternal age and educational level, in years.

Model 3 - Adjusted for maternal age (in years), maternal educational level (in years), single mother household, singleton/multiple pregnancy, having children with less than 6 years of age in household and maternal history of a diagnosed mental disorder.

^a^ Includes socialization problems, anxiety problems, depression, bipolar disorder, fear, lack of self-confidence and shyness.

**Supplemental table 2.** Associations between child neurodevelopmental or behavioural problems and maternal unemployment at child age 7 and 10 years after excluding mothers who were unemployed at baseline (prevalence ratio, PR, and respective 95% confidence interval, CI)

|  |  | **Model 1** | | | **Model 2** | | **Model 3** | | | | **Model 3** |
| --- | --- | --- | --- | --- | --- | --- | --- | --- | --- | --- | --- |
|  | **N (%)**  (n=4759) | **Child age 7** | **Child age 10** | **Child age 7** | | **Child age 10** | | **Child age 7** | **Child age 10** | **7 and 10** | |
| **Suspected** |  |  |  |  | |  | |  |  |  | |
| **Learning problems** |  |  |  |  | |  | |  |  |  | |
| No | 4337 (91.3) | 1 | 1 | 1 | | 1 | | 1 | 1 | 1 | |
| Yes | 415 (8.7) | 1.68 (1.39-2.02) | 1.74 (1.43-2.11) | 1.39 (1.15-1.67) | | 1.39 (1.15-1.69) | | 1.34 (1.11-1.61) | 1.35 (1.11-1.65) | 1.47 (1.10-1.96) | |
| **Attention problems** |  |  |  |  | |  | |  |  |  | |
| No | 3762 (79.3) | 1 | 1 | 1 | | 1 | | 1 | 1 | 1 | |
| Yes | 983 (20.7) | 1.17 (1.00-1.36) | 1.00 (0.84-1.19) | 1.07 (0.92-1.25) | | 0.93 (0.78-1.10) | | 1.06 (0.91-1.24) | 0.92 (0.78-1.10) | 1.14 (0.89-1.45) | |
| **Language problems** |  |  |  |  | |  | |  |  |  | |
| No | 4024 (84.7) | 1 | 1 | 1 | | 1 | | 1 | 1 | 1 | |
| Yes | 725 (15.3) | 1.08 (0.90-1.30) | 1.17 (0.97-1.41) | 1.01 (0.85-1.21) | | 1.08 (0.90-1.29) | | 1.01 (0.85-1.21) | 1.07 (0.89-1.29) | 1.00 (0.76-1.33) | |
| **Externalizing behaviours** |  |  |  |  | |  | |  |  |  | |
| No | 4226 (89.2) | 1 | 1 | 1 | | 1 | | 1 | 1 | 1 | |
| Yes | 514 (10.8) | 1.34 (1.11-1.62) | 1.44 (1.18-1.74) | 1.17 (0.97-1.42) | | 1.25 (1.04-1.51) | | 1.14 (0.94-1.38) | 1.19 (0.98-1.44) | 1.37 (1.03-1.81) | |
| **Developmental delay** |  |  |  |  | |  | |  |  |  | |
| No | 4634 (97.8) | 1 | 1 | 1 | | 1 | | 1 | 1 | 1 | |
| Yes | 104 (2.2) | 2.06 (1.53-2.77) | 1.97 (1.43-2.73) | 1.72 (1.28-2.32) | | 1.59 (1.13-2.22) | | 1.69 (1.26-2.28) | 1.50 (1.07-2.11) | 1.89 (1.21-2.95) | |
| **Autism** |  |  |  |  | |  | |  |  |  | |
| No | 4701 (99.2) | 1 | 1 | 1 | | 1 | | 1 | 1 | 1 | |
| Yes | 37 (0.8) | 1.42 (0.77-2.63) | 1.36 (0.70-2.66) | 1.62 (0.88-2.98) | | 1.56 (0.78-3.12) | | 1.55 (0.84-2.87) | 1.50 (0.75-2.99) | 2.27 (0.98-5.27) | |
| **Other problems^a^** |  |  |  |  | |  | |  |  |  | |
| No | 4474 (94.2) | 1 | 1 | 1 | | 1 | | 1 | 1 | 1 | |
| Yes | 274 (5.8) | 1.01 (0.76-1.34) | 1.03 (0.76-1.39) | 1.00 (0.75-1.33) | | 1.02 (0.75-1.37) | | 0.98 (0.73-1.31) | 0.97 (0.71-1.32) | 0.89 (0.55-1.42) | |
| **Diagnosed** |  |  |  |  | |  | |  |  |  | |
| **Learning problems** |  |  |  |  | |  | |  |  |  | |
| No | 4571 (96.3) | 1 | 1 | 1 | | 1 | | 1 | 1 | 1 | |
| Yes | 176 (3.7) | 1.77 (1.37-2.29) | 1.73 (1.31-2.28) | 1.49 (1.16-1.92) | | 1.41 (1.07-1.86) | | 1.46 (1.13-1.87) | 1.35 (1.02-1.79) | 1.51 (1.01-2.24) | |
| **Attention problems** |  |  |  |  | |  | |  |  |  | |
| No | 4509 (95.2) | 1 | 1 | 1 | | 1 | | 1 | 1 | 1 | |
| Yes | 228 (4.8) | 1.16 (0.87-1.55) | 1.21 (0.90-1.64) | 1.05 (0.79-1.38) | | 1.08 (0.80-1.44) | | 1.02 (0.77-1.35) | 1.03 (0.76-1.39) | 1.25 (0.84-1.87) | |
| **Language problems** |  |  |  |  | |  | |  |  |  | |
| No | 4222 (89.1) | 1 | 1 | 1 | | 1 | | 1 | 1 | 1 | |
| Yes | 519 (10.9) | 0.99 (0.80-1.23) | 1.14 (0.92-1.42) | 0.93 (0.75-1.15) | | 1.05 (0.85-1.30) | | 0.93 (0.75-1.15) | 1.03 (0.83-1.27) | 0.86 (0.61-1.22) | |
| **Externalizing behaviours** |  |  |  |  | |  | |  |  |  | |
| No | 4576 (96.8) | 1 | 1 | 1 | | 1 | | 1 | 1 | 1 | |
| Yes | 149 (3.2) | 1.20 (0.85-1.70) | 1.37 (0.97-1.92) | 1.03 (0.73-1.45) | | 1.16 (0.84-1.61) | | 0.99 (0.70-1.39) | 1.09 (0.78-1.52) | 1.40 (0.89-2.20) | |
| **Developmental delay** |  |  |  |  | |  | |  |  |  | |
| No | 4658 (98.4) | 1 | 1 | 1 | | 1 | | 1 | 1 | 1 | |
| Yes | 78 (1.6) | 2.14 (1.54-2.97) | 2.06 (1.44-2.96) | 1.75 (1.26-2.44) | | 1.62 (1.11-3.36) | | 1.72 (1.24-2.39) | 1.52 (1.04-2.23) | 1.87 (1.13-3.09) | |
| **Autism** |  |  |  |  | |  | |  |  |  | |
| No | 4718 (99.6) | 1 | 1 | 1 | | 1 | | 1 | 1 | 1 | |
| Yes | 18 (0.4) | 0.73 (0.20-2.69) | 1.20 (0.43-3.38) | 0.82 (0.23-2.98) | | 1.34 (0.46-3.87) | | 0.77 (0.21-2.80) | 1.29 (0.45-3.71) | 1.80 (0.48-6.70) | |
| **Other problems^a^** |  |  |  |  | |  | |  |  |  | |
| No | 4673 (98.5) | 1 | 1 | 1 | | 1 | | 1 | 1 | 1 | |
| Yes | 72 (1.5) | 1.00 (0.58-1.74) | 1.10 (0.64-1.91) | 1.07 (0.62-1.85) | | 1.18 (0.69-2.02) | | 1.01 (0.59-1.76) | 1.01 (0.57-1.80) | 1.04 (0.45-2.40) | |
| **Nr. of suspected problems** |  |  |  |  | |  | |  |  |  | |
| 0 | 2955 (63.1) | 1 | 1 | 1 | | 1 | | 1 | 1 | 1 | |
| 1 | 1012 (21.6) | 1.15 (0.98-1.36) | 1.16 (0.97-1.38) | 1.08 (0.92-1.27) | | 1.09 (0.92-1.29) | | 1.09 (0.93-1.29) | 1.10 (0.93-1.30) | 1.15 (0.89-1.50) | |
| 2+ | 716 (15.3) | 1.34 (1.12-1.60) | 1.33 (1.11-1.61) | 1.15 (0.97-1.37) | | 1.13 (0.94-1.36) | | 1.12 (0.94-1.33) | 1.10 (0.91-1.32) | 1.23 (0.93-1.61) | |
| **Nr. of diagnosed problems** |  |  |  |  | |  | |  |  |  | |
| 0 | 3966 (85.2) | 1 | 1 | 1 | | 1 | | 1 | 1 | 1 | |
| 1 | 441 (9.5) | 0.86 (0.67-1.11) | 1.14 (0.90-1.44) | 0.83 (0.65-1.06) | | 1.08 (0.87-1.36) | | 0.83 (0.65-1.06) | 1.08 (0.86-1.35) | 0.75 (0.50-1.13) | |
| 2+ | 249 (5.3) | 1.26 (0.97-1.64) | 1.31 (0.99-1.73) | 1.09 (0.84-1.41) | | 1.10 (0.83-1.45) | | 1.06 (0.81-1.37) | 1.05 (0.79-1.39) | 1.20 (0.81-1.76) | |

Model 1 - crude associations

Model 2 - Adjusted for maternal age and educational level, in years.

Model 3 - Adjusted for maternal age (in years), maternal educational level (in years), single mother household, singleton/multiple pregnancy, having children with less than 6 years of age in household and maternal history of a diagnosed mental disorder.

^a^ Includes socialization problems, anxiety problems, depression, bipolar disorder, fear, lack of self-confidence and shyness.

**Supplemental table 3.** Associations between child neurodevelopmental or behavioural problems and maternal unemployment at child age 7 and 10 years after excluding mothers of children with an organic disease diagnosis (prevalence ratio, PR, and respective 95% confidence interval, CI)

|  |  | **Model 1** | | | **Model 2** | | **Model 3** | | | **Model 3** |
| --- | --- | --- | --- | --- | --- | --- | --- | --- | --- | --- |
|  | **N (%)**  (n=4467) | **Child age 7** | **Child age 10** | **Child age 7** | | **Child age 10** | | **Child age 7** | **Child age 10** | **7 and 10** |
| **Suspected** |  |  |  |  | |  | |  |  |  |
| **Learning problems** |  |  |  |  | |  | |  |  |  |
| No | 4085 (91.6) | 1 | 1 | 1 | | 1 | | 1 | 1 | 1 |
| Yes | 375 (8.4) | 1.51 (1.26-1.82) | 1.60 (1.32-1.93) | 1.24 (1.03-1.48) | | 1.30 (1.08-1.57) | | 1.20 (1.00-1.44) | 1.25 (1.03-1.52) | 1.33 (1.01-1.75) |
| **Attention problems** |  |  |  |  | |  | |  |  |  |
| No | 3523 (79.1) | 1 | 1 | 1 | | 1 | | 1 | 1 | 1 |
| Yes | 930 (20.9) | 1.09 (0.94-1.27) | 1.03 (0.88-1.21) | 1.01 (0.87-1.17) | | 0.97 (0.83-1.13) | | 1.01 (0.87-1.17) | 0.96 (0.82-1.13) | 1.10 (0.88-1.39) |
| **Language problems** |  |  |  |  | |  | |  |  |  |
| No | 3773 (84.6) | 1 | 1 | 1 | | 1 | | 1 | 1 | 1 |
| Yes | 687 (15.4) | 1.20 (1.02-1.41) | 1.27 (1.08-1.50) | 1.12 (0.95-1.30) | | 1.18 (1.00-1.38) | | 1.12 (0.96-1.31) | 1.17 (1.00-1.38) | 1.17 (0.92-1.50) |
| **Externalizing behaviours** |  |  |  |  | |  | |  |  |  |
| No | 3978 (89.5) | 1 | 1 | 1 | | 1 | | 1 | 1 | 1 |
| Yes | 469 (10.5) | 1.31 (1.09-1.56) | 1.54 (1.29-1.83) | 1.15 (0.96-1.38) | | 1.37 (1.15-1.63) | | 1.13 (0.94-1.36) | 1.32 (1.11-1.58) | 1.29 (0.98-1.68) |
| **Developmental delay** |  |  |  |  | |  | |  |  |  |
| No | 4372 (98.3) | 1 | 1 | 1 | | 1 | | 1 | 1 | 1 |
| Yes | 75 (1.7) | 1.89 (1.36-2.61) | 2.28 (1.69-3.08) | 1.55 (1.12-2.13) | | 1.85 (1.34-2.55) | | 1.55 (1.13-2.13) | 1.78 (1.28-2.48) | 1.52 (0.90-2.59) |
| **Autism** |  |  |  |  | |  | |  |  |  |
| No | 4404 (99.1) | 1 | 1 | 1 | | 1 | | 1 | 1 | 1 |
| Yes | 38 (0.9) | 1.33 (0.75-2.36) | 2.07 (1.32-3.23) | 1.49 (0.84-2.63) | | 2.29 (1.42-3.69) | | 1.45 (0.81-2.58) | 2.21 (1.35-3.61) | 1.74 (0.74-4.10) |
| **Other problems^a^** |  |  |  |  | |  | |  |  |  |
| No | 4194 (94.1) | 1 | 1 | 1 | | 1 | | 1 | 1 | 1 |
| Yes | 262 (5.9) | 1.14 (0.89-1.46) | 1.10 (0.85-1.44) | 1.11 (0.87-1.43) | | 1.08 (0.83-1.40) | | 1.10 (0.85-1.41) | 1.03 (0.79-1.35) | 1.06 (0.72-1.58) |
| **Diagnosed** |  |  |  |  | |  | |  |  |  |
| **Learning problems** |  |  |  |  | |  | |  |  |  |
| No | 4318 (96.9) | 1 | 1 | 1 | | 1 | | 1 | 1 | 1 |
| Yes | 138 (3.1) | 1.48 (1.11-1.98) | 1.74 (1.32-2.28) | 1.23 (0.94-1.62) | | 1.44 (1.11-1.89) | | 1.22 (0.93-1.59) | 1.39 (1.06-1.82) | 1.28 (0.84-1.94) |
| **Attention problems** |  |  |  |  | |  | |  |  |  |
| No | 4256 (95.7) | 1 | 1 | 1 | | 1 | | 1 | 1 | 1 |
| Yes | 189 (4.3) | 1.04 (0.76-1.41) | 1.39 (1.06-1.82) | 0.93 (0.70-1.25) | | 1.26 (0.97-1.65) | | 0.93 (0.70-1.25) | 1.23 (0.94-1.62) | 1.24 (0.83-1.85) |
| **Language problems** |  |  |  |  | |  | |  |  |  |
| No | 3986 (89.6) | 1 | 1 | 1 | | 1 | | 1 | 1 | 1 |
| Yes | 465 (10.4) | 1.05 (0.85-1.28) | 1.28 (1.06-1.55) | 0.98 (0.80-1.19) | | 1.19 (0.98-1.43) | | 0.98 (0.81-1.19) | 1.17 (0.97-1.42) | 0.98 (0.72-1.33) |
| **Externalizing behaviours** |  |  |  |  | |  | |  |  |  |
| No | 4313 (97.3) | 1 | 1 | 1 | | 1 | | 1 | 1 | 1 |
| Yes | 120 (2.7) | 1.17 (0.82-1.67) | 1.46 (1.06-2.02) | 1.03 (0.73-1.46) | | 1.31 (0.96-1.79) | | 1.01 (0.71-1.43) | 1.24 (0.90-1.72) | 1.16 (0.69-1.93) |
| **Developmental delay** |  |  |  |  | |  | |  |  |  |
| No | 4391 (98.7) | 1 | 1 | 1 | | 1 | | 1 | 1 | 1 |
| Yes | 56 (1.3) | 1.92 (1.32-2.78) | 1.95 (1.32-2.87) | 1.54 (1.07-2.20) | | 1.53 (1.01-2.31) | | 1.54 (1.08-2.20) | 1.43 (0.93-2.20) | 1.45 (0.78-2.69) |
| **Autism** |  |  |  |  | |  | |  |  |  |
| No | 4420 (99.5) | 1 | 1 | 1 | | 1 | | 1 | 1 | 1 |
| Yes | 20 (0.5) | 0.84 (0.30-2.38) | 2.11 (1.16-3.84) | 0.87 (0.31-2.47) | | 2.15 (1.14-4.04) | | 0.86 (0.30-2.47) | 2.10 (1.07-4.12) | 1.20 (0.31-4.73) |
| **Other problems^a^** |  |  |  |  | |  | |  |  |  |
| No | 4379 (98.3) | 1 | 1 | 1 | | 1 | | 1 | 1 | 1 |
| Yes | 74 (1.7) | 1.14 (0.72-1.79) | 1.30 (0.84-2.02) | 1.17 (0.75-1.84) | | 1.36 (0.88-2.10) | | 1.15 (0.73-1.81) | 1.25 (0.79-1.99) | 1.03 (0.48-2.23) |
| **Nr. of suspected problems** |  |  |  |  | |  | |  |  |  |
| 0 | 2762 (63.0) | 1 | 1 | 1 | | 1 | | 1 | 1 | 1 |
| 1 | 952 (21.7) | 1.11 (0.95-1.30) | 1.03 (0.87-1.22) | 1.06 (0.91-1.24) | | 1.00 (0.85-1.18) | | 1.07 (0.92-1.25) | 1.01 (0.86-1.19) | 1.12 (0.88-1.43) |
| 2+ | 671 (15.3) | 1.36 (1.15-1.60) | 1.41 (1.19-1.66) | 1.17 (0.99-1.37) | | 1.22 (1.03-1.44) | | 1.15 (0.98-1.35) | 1.19 (1.00-1.41) | 1.29 (1.01-1.66) |
| **Nr. of diagnosed problems** |  |  |  |  | |  | |  |  |  |
| 0 | 3739 (85.8) | 1 | 1 | 1 | | 1 | | 1 | 1 | 1 |
| 1 | 415 (9.5) | 0.86 (0.68-1.09) | 1.12 (0.90-1.40) | 0.83 (0.66-1.04) | | 1.08 (0.87-1.34) | | 0.83 (0.66-1.04) | 1.07 (0.86-1.32) | 0.71 (0.48-1.04) |
| 2+ | 205 (4.7) | 1.31 (1.01-1.69) | 1.55 (1.21-1.98) | 1.12 (0.88-1.44) | | 1.33 (1.04-1.70) | | 1.11 (0.87-1.43) | 1.30 (1.01-1.67) | 1.29 (0.90-1.87) |

Model 1 - crude associations

Model 2 - Adjusted for maternal age and educational level, in years.

Model 3 - Adjusted for maternal age (in years), maternal educational level (in years), single mother household, singleton/multiple pregnancy, having children with less than 6 years of age in household and maternal history of a diagnosed mental disorder.

^a^ Includes socialization problems, anxiety problems, depression, bipolar disorder, fear, lack of self-confidence and shyness.
